# Effect of the retention ring-assisted continuous application of riboflavin in pulsed-light accelerated corneal collagen cross-linking on the progression of keratoconus

**DOI:** 10.1186/s12886-019-1085-2

**Published:** 2019-03-11

**Authors:** Chung Young Kim, Mee Kum Kim

**Affiliations:** 1 Department of Ophthalmology, Seoul National University Hospital, 101 Daehak-ro, Jongno-gu, Seoul, 110-744 South Korea; 2 Laboratory of Ocular Regenerative Medicine and Immunology, Biomedical Research Institute, Seoul National University Hospital, 101 Daehak-ro, Jongno-gu, Seoul, 110-744 South Korea

**Keywords:** Cornea, Keratoconus, Corneal cross-linking (CXL), Accelerated cross-linking, Pulsed-light, Retention ring, Riboflavin

## Abstract

**Background:**

To investigate the efficacy and safety of the retention ring-assisted continuous application of 0.1% riboflavin in pulsed-light accelerated corneal collagen cross-linking on the progression of keratoconus.

**Methods:**

The medical records of 20 eyes of 18 patients with progressive keratoconus who received collagen cross-linking at Seoul National University Hospital were retrospectively reviewed**.** Isotonic 0.1% riboflavin was continuously applied for 10 min using an 8.0-mm retention ring before the irradiation and accelerated cross-linking was applied with 30-mW pulsed-ultraviolet light at a wavelength 365 nm for eight minutes (1 s on/1 s off; 30 mW/cm^2^, cumulative dose of 7 .2J/cm^2^) without further intermittent application of riboflavin. Visual acuity, refractive error, topographic index, corneal thickness, and endothelial cell density were evaluated before the operation and at 1, 3, 6, and 12 months.

**Results:**

The best corrected visual acuity in logMAR improved from preoperative 0.43 to 0.17 in 12 months (*p* = 0.050). Maximum keratometry decreased from 51.8 D to 50.4 D at 6 months (*p* = 0.015) and 50.1 D at 12 months (*p* = 0.0003). Astigmatism decreased from preoperative 5.5 D to 4.1 D at 12 months (*p* < 0.0001). Thinnest corneal thickness decreased at three and 6 months but recovered in 12 months (*p* > 0.05). Endothelial cell density decreased at postoperative 1 month (*p* = 0.02) but gradually recovered in 12 months (*p* > 0.05).

**Conclusions:**

Retention ring-assisted continuous application of riboflavin for 10 minutes in pulsed-light accelerated cross-linking is a comparably safe and effective treatment for halting the progression of keratoconus in 12 months when compared to outcomes of the standard Dresden protocol shown in previous reports.

**Electronic supplementary material:**

The online version of this article (10.1186/s12886-019-1085-2) contains supplementary material, which is available to authorized users.

## Background

Keratoconus is an ectatic corneal disorder characterized by progressive thinning and protrusion, accompanied by irregular astigmatism and myopic progression, thereby deteriorating visual function. The conventional standardized treatment is keratoplasty in advanced keratoconus [1]. Corneal collagen cross-linking has recently been investigated to intervene in progression of moderate keratoconus. In corneal cross-linking (CXL), riboflavin (vitamin B2) acts as a photosensitizer. When it is combined with ultraviolet A (UV-A) of 365-nm wavelength, oxygen radicals are produced via photochemical reaction. Oxygen radicals can introduce interfibrillar and intrafibrillar covalent bonds among collagen fibrils [2, 3]. Such chemical bonds can increase the corneal rigidity and its resistance to enzymatic degradation of keratoconic cornea [4], thereby restoring the stability of the cornea and halting the progression of keratoconus.

In the standard Dresden protocol, cornea is deepithelialized using alcohol 20% solution and soaked in isotonic 0.1% riboflavin with 20.0% dextran solution for 30 min. The cornea is then irradiated with UV-A of 3 mW/cm2 for 30 min (total energy 5 .4mJ/cm2). Riboflavin is intermittently applied before and during the irradiation [5]. This protocol can effectively stabilize the cornea of progressive keratoconus in 10 years [6]. However, it is considered time-consuming by some clinicians.

An accelerated cross-linking protocol was then introduced to shorten the procedure. It applies UV-A of greater intensity for a shorter irradiation time based on the Bunsen-Roscoe law of reciprocity [7]. According to the theory, total energy delivered to the cornea remains identical. Its biological effect is proportional to the energy delivered. Surgical procedure of accelerated protocol is basically the same as the standard Dresden protocol except two aspects; the intensity (from 9 to 30 mW/cm^2^) and irradiation time of UV-A (from 3 to 15 min) [8–16]. Many studies have reported that clinical results of the accelerated protocol are comparable to those of the standard Dresden protocol. However, combination of intensity and irradiation time is diverse. It has not been standardized yet. Furthermore, recent meta-analyses have shown that the standard Dresden protocol has better treatment efficacy in depth of demarcation line and change of keratometric values than the accelerated protocol [15, 16].

A study on photochemical kinetics of cross-linking has shown that continuous UV-A irradiation can induce rapid depletion of oxygen in riboflavin applied corneal tissue and that turning the UV off can replenish oxygen level [17]. Photodynamic reaction between riboflavin molecules and UV-A can induce release of reactive oxygen species (ROS) and introduces cross-linking in collagen fibers. According to the kinetics model, pulsing the UV-A irradiation theoretically can restart the photodynamic reaction and maintain ROS concentration in the cornea [17]. Based on these findings, pulsed-light accelerated cross-linking had been adopted to enhance treatment efficacy. It has been reported to be as effective as the standard Dresden protocol [18–20].

So far, there is no standardized method for riboflavin application in the accelerated protocol. The protocol provided by the manufacturer (Avedro. Inc., Waltham, MA, USA) recommends intermittent application of isotonic riboflavin every 2 minutes for a total of 10 minutes. Recent studies have reported different clinical efficacies with various application time (from 10 to 30 min) and intervals (2 to 5 minutes) [9–12, 19]. Shorter application time (every 2 minutes for 10 min) seems to have lower efficacy, even in pulsed-light accelerated protocol, than the standard protocol [12]. In addition, our group has recently demonstrated possible damage to the limbal stem-cell area by exposure to riboflavin during cross-linking [21]. Thus, it is necessary to protect the corneal limbal area and prevent it from exposure to riboflavin and UV.

To enhance the efficacy of cross-linking in progressive keratoconus and add limbal protection, our group adopted pulsed-light cross-linking protocol with two modifications. First, we introduced a retention ring for the application of riboflavin in order to apply riboflavin continuously (10 min) for better penetration into the cornea while enhancing the efficacy and reducing exposure of limbal area to riboflavin. Second, we applied a trephined silicone hydrogel bandage contact lens on limbal area during irradiation of UV-A to protect it from toxic effect of UV [21]. UV-A was irradiated in a pulsatile mode (1 s on and off) for a total of 8 min with total energy of 7 .2J/cm^2^ as described in a previous report [18]. We hypothesized that this novel method of retention ring-assisted continuous application of 0.1% riboflavin for 10 minutes in pulsed-light accelerated cross-linking might have better or comparable efficacy and safety profile for halting the progression of keratoconus than other pulsed modes, accelerated cross-linking protocol, or standard Dresden protocol described in previous reports.

## Methods

### Patients

This study was approved by the Institutional Review Board of Seoul National University College of Medicine (IRB No. 1807–139-961, Seoul, South Korea) and adhered to the Declaration of Helsinki. It included 20 eyes of 18 patients with progressive keratoconus who received CXL at a single institution (Seoul National University Hospital) by a single surgeon (M.K. Kim). The medical records of the 18 patients (14 male and 4 female patients) were retrospectively reviewed. The mean age was 28.0 ± 7.3 (range 17–43) years, and all patients were treated with the same protocol.

### Inclusion and exclusion criteria

The patients who were diagnosed with progressive keratoconus and underwent accelerated CXL were included in the study. Progression was confirmed by an increase of maximum keratometry (*K*_max_) of more than 1.5 diopters (D) / year in serial topography. Those with preoperative *K*_max_ greater than 60 D or central corneal thickness (CCT) less than 400 μm or thinnest corneal thickness (TCT) less than 390 μm were excluded. Patients combined with other ocular surface diseases were also excluded.

### Clinical evaluation

Preoperative and postoperative examinations included best-corrected visual acuities (BCVA) as a logarithm of the minimum angle of resolution (logMAR), refractive errors by Auto Kerato-Refractometer (KR-8900, Topcon, Tokyo, Japan), keratometric values including maximum (*K*_max_), minimum (*K*_min_), average (*K*_avg_), and 3 mm and 5 mm irregular index by topography (ORBSCAN IIz, Technolas Perfect Vision GmbH, München, Germany). Corneal thickness (CT) was *measured* by anterior segment optical coherence tomography (AS-OCT, Visante OCT; Carl Zeiss Meditec, Dublin, CA). Noncontact specular microscopy (SP-8800, Konan, Hyogo, Japan) was used to measure endothelial cell density. Preoperative measurements were compared with the postoperative measurements at 1, 3, 6, 12 months.

### Surgical procedure

Eyes were anesthetized with topical 1% proparacaine (Alcaine; Alcon, Fort Worth, TX) and the central 9.0-mm corneal epithelium was peeled off using a crescent knife (Beaver; Beaver-Visitec, Waltham, MA). Intraoperative pachymetry (Pocket II; Quantel Medical, Bozeman, MT) was performed to make sure that the corneal thickness was greater than 325 μm. A retention ring 8.0 mm in diameter (Frimen, Inc., Jiangsu, China) was applied on the epi-off corneal surface, and 0.1% isotonic riboflavin (Vibex Rapid, Avedro, Inc., Waltham, MA, USA) with dextran-free hydroxypropyl methylcellulose (HPMC; VibeX Rapid, Avedro, Waltham, MA) was continuously applied for 10 minutes within the retention ring (Additional file 1: Figure S1 A). Then, a trephined (inner diameter, 8.5 mm) silicone hydrogel bandage contact lens (ACUVUE OASYS; Johnson & Johnson Vision Care, Jacksonville, FL) was applied to cover the limbus from UV irradiation (Additional file 1:Figure S1 B and C). Pulsing (1 s. on/off) 30 mW/cm^2^ intensity of 365-nm wavelength UV-A (Avedro, Waltham, MA) was irradiated for 8 minutes, resulting in a cumulative dose of 7 .2J/cm^2^. During the irradiation, riboflavin was not applied. After irradiation, the corneal surface was irrigated with a balanced salt solution, and the silicone hydrogel bandage contact lens was applied. The bandage contact lens was maintained for 7 days with topical 0.5% moxifloxacin and 1% prednisolone four times a day.

### Statistical analysis

Statistical analysis was performed using Prism 5 software (Graphpad Software, Inc., San Diego, CA). The two-tailed Student’s *t* test was used to compare each of the baseline parameters with the following measurement. The data are presented with mean value ± mean standard deviation (SD), and statistical significance was verified if *p* < 0.05.

## Results

This study analyzed a total of 20 eyes of 18 patients over a mean postoperative follow-up of 12.7 ± 1.5 months. The demographics and baseline characteristics of the study group are shown in Table 1. The mean age at the time of the procedure was 28.0 ± 7.3 years. Mean depth of the demarcation line was 281.0 ± 39.9 μm (minimum 210 μm to maximum 380.0 μm) (Additional file 2: Figure S2, Table 1). The penetration depth (demarcation line) in our study is comparable to or deeper than that in other studies on standard Dresden protocol or accelerated cross-linking protocols (Table 2).

**Table 1:** Baseline characteristics of the patients who underwent pulsed accelerated cross-linking in this study

Demographic factors	
Eyes	20 eyes of 18 patients
Sex	14 male (77.7%), 4 female (22.3%)
Age (year)	28.0 ± 7.3
Systemic disease / Atopy	0 (0%) / 2 (11%)
FU (month)	61.3 ± 53.6
POD (month)	12.8 ± 1.0
BCVA (logMAR)	0.43 ± 0.43
UCVA (logMAR)	0.81 ± 0.38
Refractive error	
Spherical (diopter)	−7.40 ± 2.80
Cylinder (diopter)	−4.81 ± 1.36
SE (diopter)	−9.81 ± 2.93
Topography	
Kmax (diopter)	52.20 ± 4.32
Kmin (diopter)	46.50 ± 2.18
Kavg (diopter)	49.35 ± 3.06
Astigmatism	5.71 ± 3.07
IR, 3 mm	6.38 ± 2.17
IR, 5 mm	6.23 ± 1.86
AS-OCT	
CCT (μm)	492.1 ± 39.1
TCT (μm)	466.6 ± 37.1
ECD (cells/ mm^2^)	2731 ± 279.5
Depth of demarcation line after 1mo of cross-linking (μm)	281.0 ± 39.9

Values are presented as mean ± SD, FU, follow up; POD, postoperative date; BCVA, best-corrected visual acuity; UCVA, uncorrected visual acuity; logMAR, logarithm of minimum angle of resolution; SE, spherical equivalent; IR, irregular astigmatism; CCT, central corneal thickness; TCT, thinnest corneal thickness; AS-OCT, anterior segment optical coherence tomography; ECD, endothelial cell density

**Table 2:** Comparison of the application method of UV / riboflavin and parameter values in standard Dresden and accelerated cross-linking of previous studies. The first three studies used pulsed UV irradiation, while the others used continuous UV

	Irradiation^*^	Total Dose^*^ (J/cm^2^)	Riboflavin application^*^: before/during irradiation	FU (mo.)	Standard Dresden Protocol						Accelerated Protocol					
					N	Δ BCVA (logMAR)	Δ Kmax (diopter)	Δ CCT (μm)	Δ ECD (cell/cm^2^)	DDL (μm)	N	Δ BCVA (logMAR)	Δ Kmax (diopter)	Δ CCT (μm)	Δ ECD (cell/cm^2^)	DDL (μm)
Our study	30 mW/cm^2^ for 8 min (1 s. on/off)	7.2	with HMPC for 10 min, ontinuous / Not applied	12	Not available						20	−0.25	−1.54	− 10.3	−91	281
Mazzotta [20]	15 mW/cm^2^ for 12 min (1 s. on/off)	5.4	with HMPC every 1 min for 10 min / NR	24	Not available						132	− 0.14	−0.42	5.03	NR	280
Jiang [19]	30 mW/cm^2^ for 8 min (1 s. on/off)	7.2	with HMPC every 2 min for 10 min / every 3 min	12	31	− 0.12	−1.80	NR	− 109	285	36	−0.09	−1.31	NR	−247	202
Bouheraoua [24]	30 mW/cm^2^ for 3 min	5.4	20% dextran every 1 min for 10 min / NR	6	15	−0.05	−1.80	−1	+ 4	303	15	−0.01	0.50	−3	20	184
Brittingham [25]	9 mW/cm^2^ for 10 min	5.4	20% dextran every 5 min for 20 min / every 2 min	12	81	NR	−0.76	NR	NR	323	50	NR	0.72	NR	NR	245
Hagem [26]	9 mW/cm^2^ for 10 min	5.4	with HPMC for 20 min / every 2 min	12	20	−0.11	−1.40	NR	− 112	442	20	−0.09	−0.50	NR	−54	317
Ng [27]	9 mW/cm^2^ for 10 min	5.4	20% dextran every 2 min for 25 min / every 5 min	13.9	14	−0.13	−1.80	−2.1	NR	283	12	0.02	−0.30	2.1	NR	209
Shetty [8]	18 mW/cm^2^ for 5 min	5.4	20% dextran every 2 min for 30 min / every 2 min	15.3	36	0.04 (decimal)	−1.32	NR	− 166	280	33	0.10 (decimal)	−0.52	NR	− 162	203

*Irradiation and riboflavin application method of accelerated protocol, Values are presented as mean value, FU, follow up; N, number; mo, months; BCVA, best-corrected visual acuity; logMAR, logarithm of minimum angle of resolution; Kmax, maximum keratometry; CCT, central corneal thickness; ECD, endothelial cell density; DDL, depth of demarcation line; NR, not reported; ΔBCVA (logMAR), BCVA at last FU - preoperative BCVA; ΔKmax, Kmax at last FU - preoperative Kmax; ΔCCT, CCT at last FU - preoperative CCT; ΔECC, ECC at last FU - preoperative ECC

**Fig. 1:**
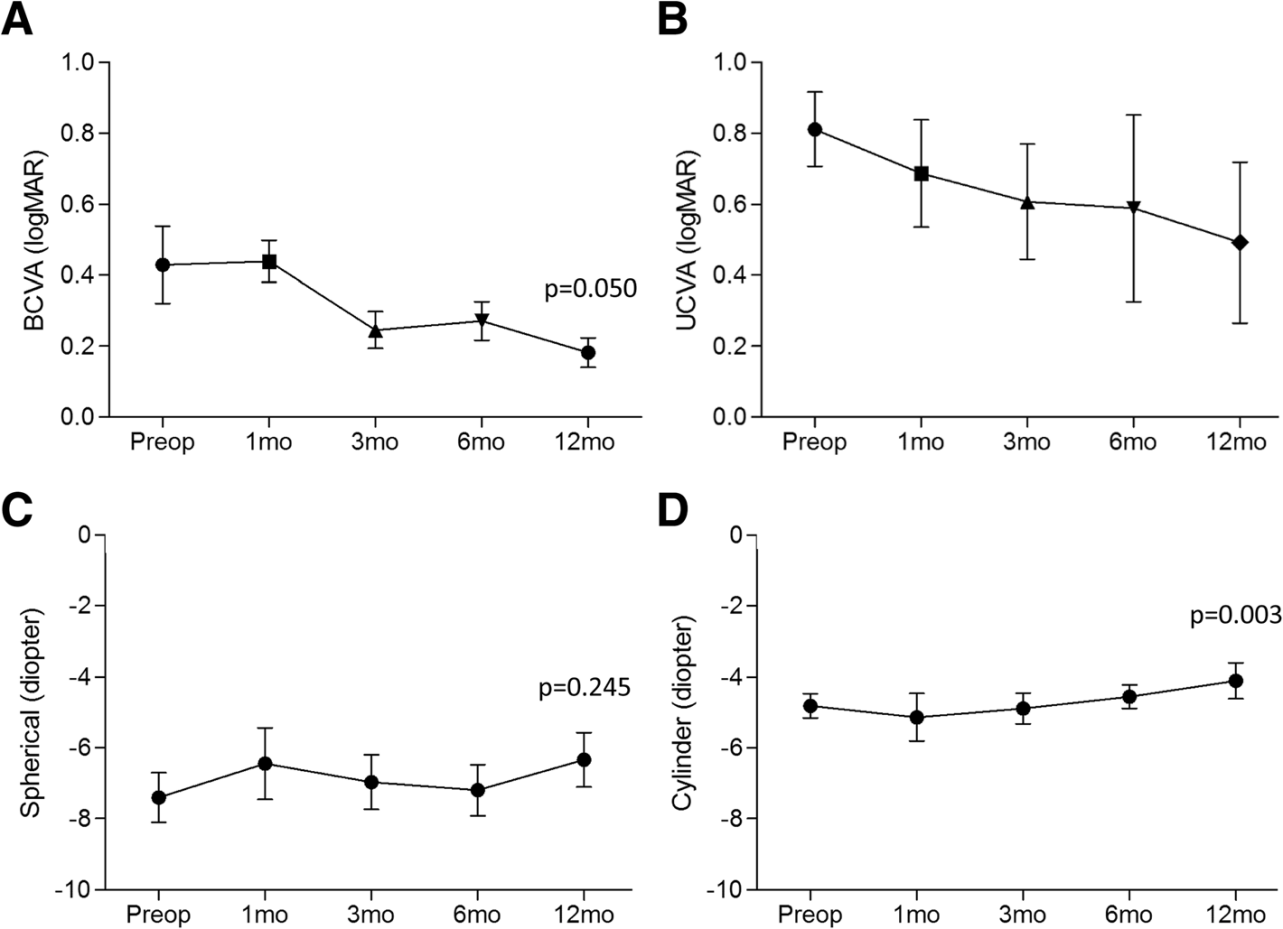
Changes of best corrected visual acuity (BCVA, logMAR) (**a**), uncorrected visual acuity (UCVA, logMAR) (**b**) and spherical (**c**), cylindrical (**d**) refractive errors (diopter) over time following modified pulsed-light accelerated cross-linking

The best corrected visual acuity gradually improved from preoperative 0.43 ± 0.43 logMAR to 0.17 ± 0.16 logMAR at 12 months with a marginal significance (*p* = 0.050) (Fig. 1a) and uncorrected visual acuity also improved, from 0.81 ± 0.38 logMAR to 0.49 ± 0.50 logMAR at 12 months, although it was not statistically significant (*p* = 0.635) (Fig. 1b). Refractive errors decreased from − 7.40 ± 2.80 D to − 6.25 ± 3.11 D in spherical (*p* = 0.245), − 4.81 ± 1.36 D to − 4.00 ± 2.07 D in cylinder (*p* = 0.003) and − 9.81 ± 2.93 D to − 8.25 ± 2.98 D in spherical equivalent (*p* = 0.075) after 12 months, and remained stable thereafter respectively) (Fig. 1c and d). Cylindrical reduction was a statistically significant with this modified protocol.

**Fig. 2:**
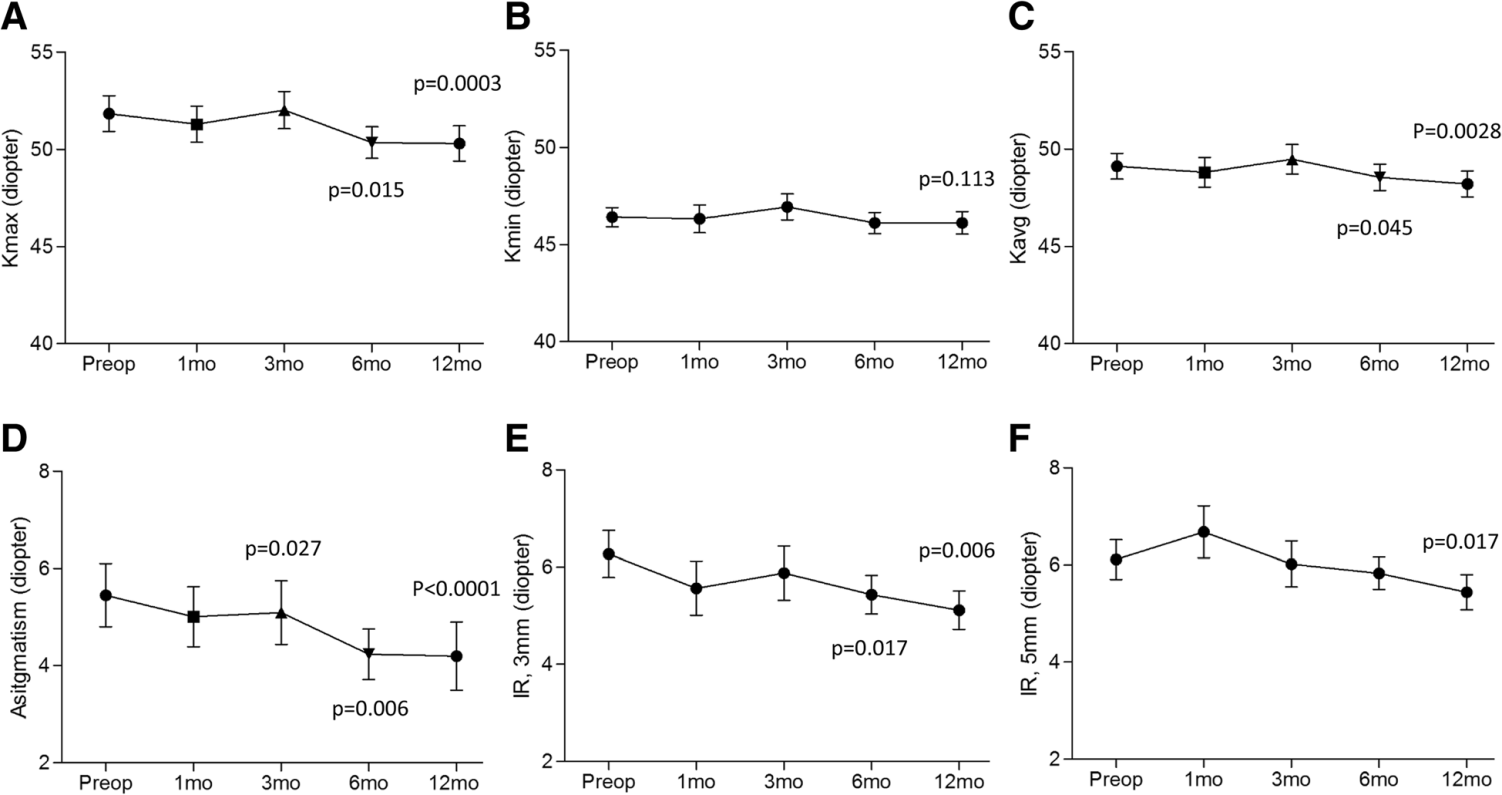
Corneal topographic changes (diopter) following modified pulsed-light accelerated cross-linking over time. *K*_max_ (**a**), *K*_min_ (**b**), *K*_avg_ (**c**), astigmatism (**d**), and irregular astigmatism (IR) at 3 mm (**e**) and at 5 mm (**f**)

Keratometric values were delineated as *K*_max_ (maximum), *K*_min_ (minimum), and *K*_avg_ (average). *K*_max_ (Fig. 2a) significantly decreased from 51.85 ± 4.11 D to 50.36 ± 3.55 D at 6 months (*p* = 0.015) and 50.11 ± 3.62 D at 12 months (*p* = 0.0003). *K*_min_ (Fig. 2b) decreased, from 46.40 ± 2.19 D to 45.99 ± 2.27 D at 12 months (*p* = 0.113) although it was insignificant. *K*_avg_ (Fig. 2c) also significanly decreased from 49.12 ± 2.95 D to 48.55 ± 2.79 D at 6 months (*p* = 0.045) and 48.05 ± 2.69 D (*p* = 0.0028) at 12 months. Topographic astigmatism was significantly reduced from preoperative 5.45 ± 2.90 D to 4.23 ± 2.27 D at 6 months (*p* = 0.006) and 4.10 ± 2.75 D at 12 months (*p* < 0.0001) (Fig. 2d). Irregular index at 3 mm and 5 mm decreased from preoperative 6.27 ± 2.16 D to 5.06 ± 1.53 D (*p* = 0.006) and 6.11 ± 1.82 D to 5.36 ± 1.38 D (*p* = 0.017), and the reductions were statistically significant at 12 months. (Fig. 2e and f).

**Fig. 3:**
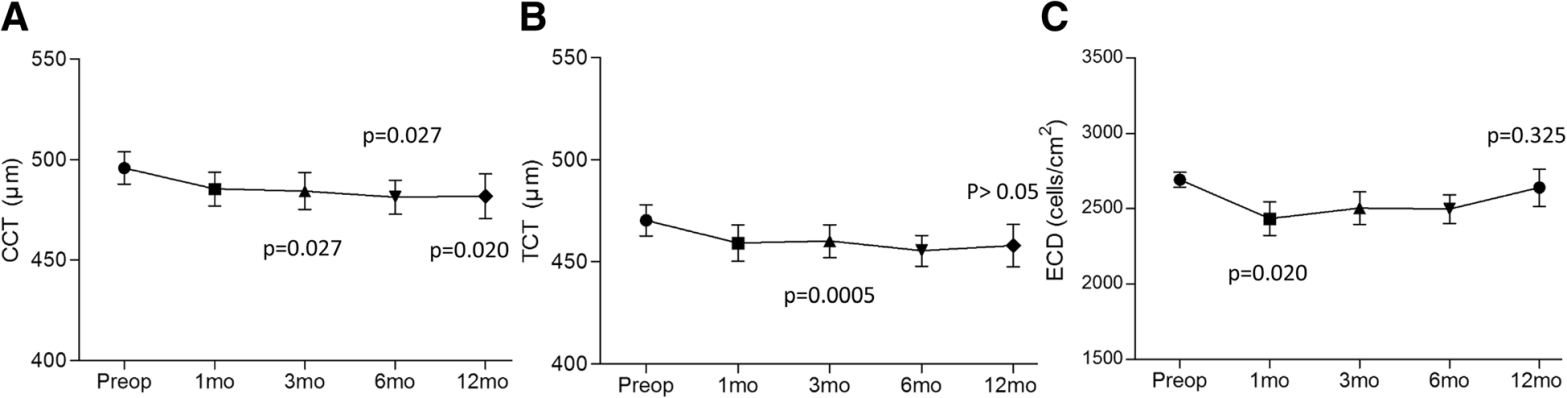
Changes of central corneal thickness (μm) (**a**), thinnest corneal thickness (μm) (**b**) and endothelial cell density (**c**) (cells/cm^2^) following modified pulsed-light accelerated cross-linking at different postoperative periods

Central corneal thickness showed a significant decrease, from 495.9 ± 36.1 μm to 484.5 ± 41.4 μm in 12 months (*p* = 0.020) (Fig. 3a). Thinnest corneal thickness was temporarily decreased at 6 months (*p* = 0.0025), but eventually recovered to 460.5 ± 38.3 μm in 12 months, resulting in no statistical difference compared with the preoperative level (*p* > 0.05, pre-operation vs 12 mo.) (Fig. 3b).

Endothelial cell density significantly decreased, from preoperative 2693 ± 223 cells/mm^2^ to 2434 ± 460 cells/mm^2^ at 1 month (*p* = 0.018), but gradually recovered back in 12 months (2621 ± 471 cells/mm^2^) without any significant change compared with preoperative level or 3 months (*p* = 0.325, preop vs 12 mo., *p* = 0.08, 3 mo. vs 12 mo.) (Fig. 3c).

There was a single case of delayed wound healing, which spontaneously resolved in 14 days with conservative treatments using preservative artificial tear; otherwise, there were no serious complications related to the procedure.

## Discussion

In this study, we report that retention ring-assisted continuous application of riboflavin for 10 minutes in pulsed-light accelerated cross-linking is as effective as standard Dresden protocol or more effective than other pulsed-light accelerated cross-linking protocols for halting the progression of keratoconus in 12 months. With regard to safety, endothelial cell density changes in 12 months of this protocol are comparable to outcomes described in previous reports (Table 2).

To date, there is no consensus on a standardized protocol for accelerated cross-linking comparable to the standard Dresden protocol, which has been documented to be effective in treating progressive keratoconus by improving corrected distance visual acuity, decreasing keratometric values, and preserving endothelial cells in a ten-year follow-up study [6]. To improve efficacy, a pulsed-light accelerated protocol was first introduced, showing better functional outcomes in 1 year by optimizing intraoperative oxygen availability than the standard Dresden protocol [18]. It also showed effectiveness in a two-year follow-up [20]. Nevertheless, recent meta-analysis studies generally indicate that the standard Dresden protocol has better efficacy than for the accelerated protocol with a deeper demarcation line [15, 16]. However, protocol modification is still required to improve outcomes of the accelerated protocol. We hypothesized that continuous application of riboflavin using a retention ring might induce deeper penetration of riboflavin and increase the availability of riboflavin in corneal tissue, thus enhancing the efficacy of treatment.

To shorten the procedure time without reducing its efficacy, we aimed to modify the application method of riboflavin by continuously using a retention ring before irradiation. The overall clinical effect of our modified protocol on visual acuity, topographic parameters and demarcation line depth was comparable to that of the standard Dresden protocol or better than other studies with accelerated cross-linking (Table 2). The depth of demarcation line in this study was the greatest (281 μm) among pulsed-light accelerated protocols reported (202 ~ 280 μm). It was comparable with other continuous accelerated protocols (184 ~ 317 μm) or the standard Dresden protocol (280 ~ 442 μm). The mean visual improvement was − 0.25 logMAR in our study, which was better than the outcome in the accelerated protocol (− 0.01 ~ − 0.14 logMAR) and the standard Dresden protocol (− 0.05 ~ − 0.13 logMAR). The mean reduction of maximum keratometry was − 1.54 D in our study. It was greater that of other accelerated protocols (− 0.3 D ~ − 1.31 D) and comparable to that of the standard Dresden protocol (− 0.76 D ~ − 1.80 D) (Table 2). Therefore, our modified ten-minute continuous application of riboflavin in a pulsed-light accelerated protocol has better efficacy than other accelerated protocols. It is as efficient as the standard Dresden protocol in halting the progression of keratoconus.

Because of the possibility of limbal stem cell damage during riboflavin application and UV irradiation [21], modification of the previous protocol as essential. In our in vivo experiments [21], a significantly greater decrease of p63^+^ stem cells was observed when riboflavin was dropped on the whole corneal surface in accelerated cross-linking when compared to the protocol using the retention ring to confine riboflavin to only the central cornea. We believe that protecting the limbal stem cells by minimizing their exposure to riboflavin and UV is important, given their crucial role in regeneration of cells. Further experimental studies on concentration- and duration-dependent toxicity of riboflavin are needed to elucidate cellular injury and standardize the protocol.

In our study, endothelial cell density was significantly reduced in postoperative 1 month compared to that pre-operatively (*p* = 0.02). It is plausible that continuous application of riboflavin may penetrate deeper microscopically than the depth that the grossly visible demarcation line indicates. This may subsequently lead to effect of UV on endothelial cells immediately post-operation. The loss of endothelial cells (− 9.61%) was not clinically significant to induce corneal edema. In addition, endothelial cell density gradually recovered and maintained up to postoperative 12 months. The overall reduction was not statistically significant (− 2.67%, preop vs 12 months). It was a comparable change (− 91 cells/cm^2^) when we compared it with previous data (− 54 ~ − 247 cells/cm^2^, Table 2). Most of previous studies including the accelerated protocol demonstrated no clinically significant reduction of ECD after 12 months (Table 2) [15, 16]. Meanwhile, one study has shown a significant reduction of ECD up to 12 months after accelerated cross-linking in keratoconus [22]. An anecdotal report has presented corneal edema after cross-linking [23]. Therefore, endothelial damage should be carefully monitored when we modify these procedures. Further study is needed to explore the most appropriate continuous application time of riboflavin with the same efficacy and enhanced safety.

In this study, two patients had corneal cross-linking bilaterally. These procedures were performed sequentially without simultaneous application. It was not an intentional intra-patient control because this was a retrospective study. In fact, we conducted cross-linking in one eye in each patient, and cross-linking improved visual acuity significantly. Consequently, these patients were very satisfied with the surgical outcome and wanted to undergo cross-linking for the other eye. Subsequently, they underwent cross-linking for the contralateral eyes.

This study was limited because it did not include a direct control group with Dresden protocol. In addition, the sample size was small. Instead, we compared the efficacy and safety of our modified protocol with those of the Dresden protocol described in previous reports (Table 2). They showed comparable outcomes. This is the first report on a novel method with clinically significant one-year follow-up outcomes.

## Conclusion

Retention ring-assisted continuous application of riboflavin for 10 minutes in pulsed-light accelerated cross-linking is a comparably effective and safe treatment for halting the progression of keratoconus in 12 months when compared to outcomes of the standard Dresden protocol described in previous reports.

## Additional files

Additional file 1: **Supplementary Figure S1.** Riboflavin is contained inside a retention ring during the application for 10 minutes (**A**). A trephined silicone hydrogel bandage contact lens is applied during UV irradiation (**B, C**) (TIF 9431 kb)

Additional file 2: **Supplementary Figure S2.** Slit-lamp biomicroscopy exam after a month of modified pulsed-light accelerated cross-linking demonstrated a demarcation line (**A**, yellow arrow). Anterior segment optical coherence tomography-assisted measurement of demarcation line depth (**B**, yellow arrow) (TIF 6419 kb)

## Abbreviations

AS-OCT: antesrior segment optical coherence tomography
BCVA: best corrected visual acuity
CCT: central corneal thickness
CXL: cross-linking
D: diopter
DDL: depth of demarcation line
ECD: endothelial cell density
FU: follow up
IR: irregular astigmatism
Kavg: average keratometric value
Kmax: maximal keratometric value
Kmin: minimal keratometric value
LogMAR: logarithm of minimal angle of resolution
POD: postoperative date
SD: standard deviation
SE: spherical equivalent
TCT: thinnest corneal thickness
UCVA: uncorrected visual acuity
UV: ultraviolet
