# Supplementary figures and images for: Effect of the retention ring-assisted continuous application of riboflavin in pulsed-light accelerated corneal collagen cross-linking on the progression of keratoconus
Source: BMC Ophthalmol. 2019 Mar 11;19:72. doi: 10.1186/s12886-019-1085-2 (PMC6417158; doi:10.1186/s12886-019-1085-2)

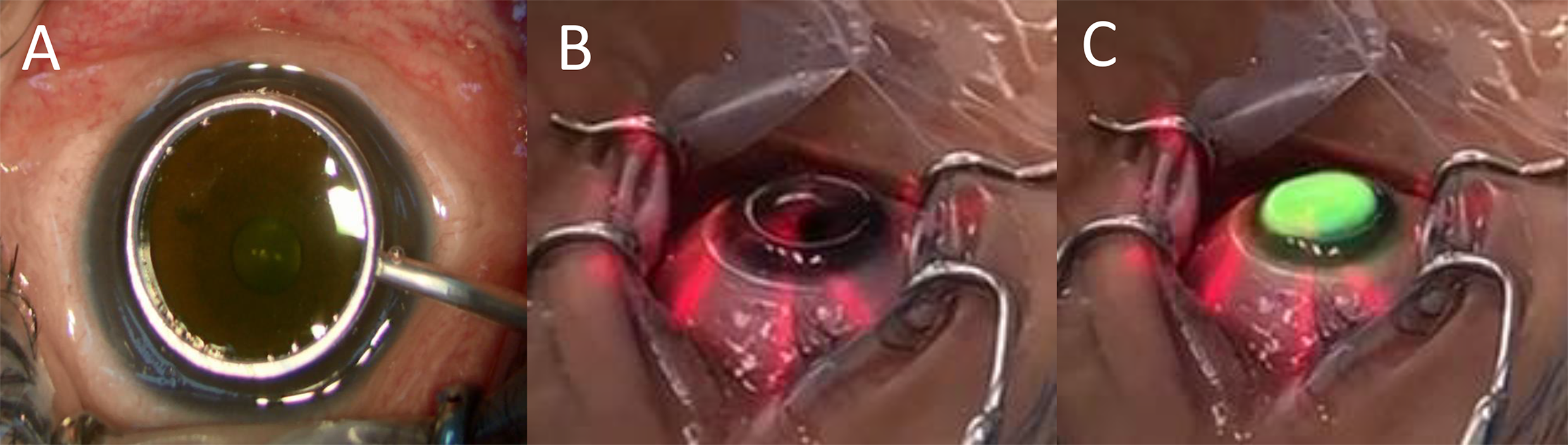

Supplement: Supplementary file 1 — Supplementary Figure S1. Riboflavin is contained inside a retention ring during the application for 10 minutes (A). A trephined silicone hydrogel bandage contact lens is applied during UV irradiation (B, C) (TIF 9431 kb) [file 12886_2019_1085_MOESM1_ESM.tif]

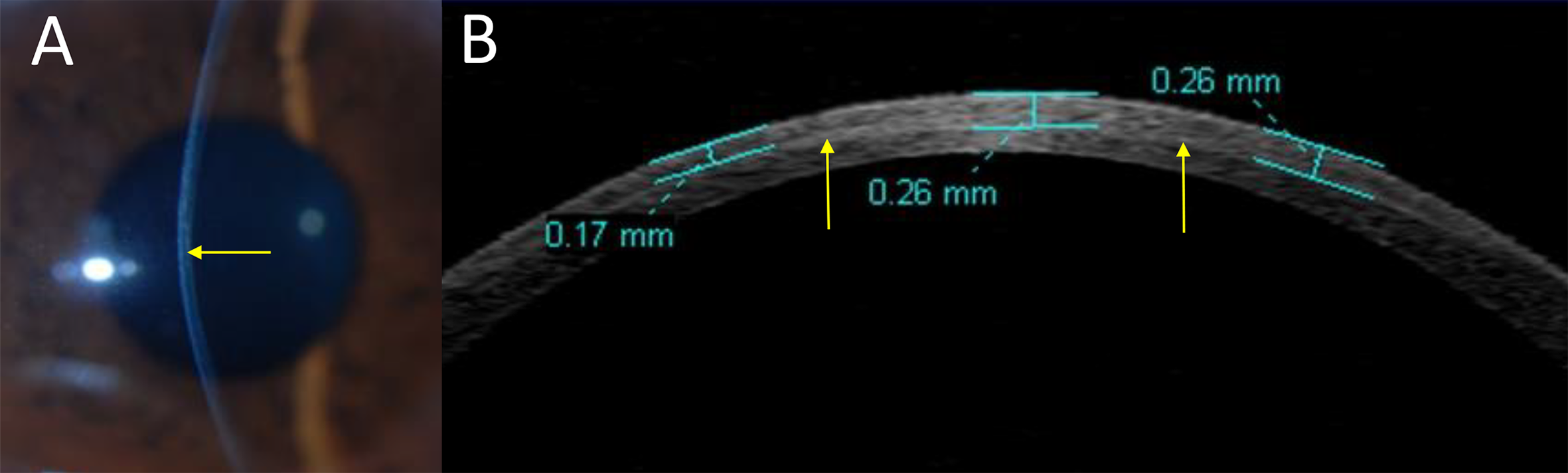

Supplement: Supplementary file 2 — Supplementary Figure S2. Slit-lamp biomicroscopy exam after a month of modified pulsed-light accelerated cross-linking demonstrated a demarcation line (A, yellow arrow). Anterior segment optical coherence tomography-assisted measurement of demarcation line depth (B, yellow arrow) (TIF 6419 kb) [file 12886_2019_1085_MOESM2_ESM.tif]
